# Comparison of the Speech Syntactic Features between Hearing-Impaired and Normal Hearing Children

**Published:** 2014-04

**Authors:** Mohammad Reza PahlavanNezhad, Hamid Tayarani Niknezhad

**Affiliations:** 1 *Faculty of Linguistics, Ferdowsi University of Mashhad, Mashhad, Iran.*

**Keywords:** Auditory-verbal therapy, Children, Hearing loss, Syntactic skills

## Abstract

**Introduction:**

The present study seeks to describe and analyze the syntactic features of children with severely hearing loss who had access to the hearing aids compared with children with normal hearing, assigning them to the same separate gender classes.

**Materials and Methods:**

In the present study, eight children with severe hearing impairment who used a hearing aid and eight hearing children matched for age and gender were selected using an available sampling method based on the principles of auditory-verbal approach. Hearing children had an average age of 5.45 ±1.9 years and subjects had a mean age of 5.43±2.17 years and their rehabilitation had begun before they were 18 months old. The assessment instrument of the study included the language development test, TOLDP-3. The syntactic skills of these children were analyzed and compared with the hearing children of the same age based on gender.

**Results:**

There was a significant difference between the syntactic scores of the hearing-impaired children and the scores of the hearing children of the same age in the “sentence imitation” (t=−2/90, P<0/05) and “grammatical completion” (t=−3/39, P<0/05) subtests, with no significant difference in the “grammatical understanding” subtest (t=1/67, P>0/05). Moreover, there was no significant difference between male and female children with hearing impairment in terms of syntactic skills development.

**Conclusion:**

With early diagnosis and timely rehabilitating intervention, children with hearing loss can perform in a similar way to children of their age with normal hearing in some syntactical areas. Furthermore, the gender factor in the present study had no effect on the development of syntactical skills of children with hearing loss.

## Introduction

Language is such a complex system that only humans have the capacity to learn it. Furthermore, language skills are acquired gradually and in specific steps. At the age of 5, a child’s linguistic system is almost identical to that of an adult to the extent that he/she can meditate and express thoughts accurately via language (1). 

The first 36 months of a child's life are critical for language learning, and are incomparable with any other periods of life in terms of language acquisition. Therefore, age is a crucial factor in the emergence and development of language (2). According to the critical period theory, the acoustic stimuli received through the auditory system are necessary for the neural growth of the auditory-perceptive areas in infants. In hearing-impaired children, due to the paucity of such stimuli, the neuroplasticity required for the development of language skills is lost (3). A perceptual pattern would suggest that the age at which a hearing aid is used is critical as it relates to sensitive periods in the development of the central auditory system. Further, the training-based pattern focuses on metalinguistic abilities which are likely to be more closely linked to general maturation and duration of language exposure (4). 

The speech of children with hearing impairment, especially those with severe hearing loss (71– 90dB threshold), is barely comprehensible; an issue that can expose these children to a plethora of social, emotional, educational, and perceptive problems (5). The results of the research by Yoshinaga-Itano et al reveal that if hearing-impaired children with natural cognitive skills are diagnosed before the age of 6 months, timely and appropriate intervention can help them develop their linguistic skills to a natural level consistent with their cognitive skills (6). Miller maintains that language acquisition is a mental function associated with the brain, suggesting that despite hearing impairment, these children have a normal brain and mind (7). 

Linguists define syntax as the organization or arrangement of the words in a sentence. In syntactic relations, two types of relations are usually mentioned: syntagmatic relations as well as hierarchical and paradigmatic relations. In a linear organization of the speech, we deal with the syntagmatic elements in a specific order, while in the hierarchical (syntagmatic) organization, the internalized structures are addressed. Both cases require grammatical knowledge, which, as pointed out by Chomsky, is innate and internalized in the human being, waiting to be recalled and utilized when the situation arises (8).

Grammatical and syntactic problems are among the linguistic issues that children with hearing loss encounter. Studies have shown that children with hearing loss pass the growth stage of language development at a slower pace compared with their peers; a subject that has been investigated in different linguistic areas including syntax, semantics, pragmatics and even writing (9). The development pattern of these linguistic skills, including syntactic and grammatical acquisition in children with hearing loss, is identical to the development pattern of children with normal hearing (10). Hearing-impaired children face many problems in terms of learning and utilizing functional and lexical morphemes such as adverbs, prepositions, and pronouns along with using relative clauses, complex sentences, and verb inflections. Hence, the scale and variety of vocabulary in children with hearing loss is limited and their utterance length is usually shorter than that of hearing children (11).

Bamford and Saunders also showed that the hearing impaired are more likely to use content words, nouns, and verbs in their speech while grammatical words such as prepositions, pronouns, auxiliary verbs and conjunctions are rarely used (12). Gray claims that the hearing-impaired face restrictions and delay in vocabulary development, with a tendency to use concrete nouns more than abstract nouns in their speech (13). In a study on vocalization and the emergence of first words in children with hearing loss who have been exposed to habilitative intervention for two years and children with normal hearing, it was revealed that vocalization increased in both groups between the age of 16 to 24 months, but the extent of the vocabulary used by children with hearing loss was significantly lower than children with normal hearing at the age of 54 months. Moreover, children with normal hearing were able to use complex vocabularies with higher clarity (14,15). There was also a direct relationship between hearing loss and the delay in linguistic aspects (16). To date, the limited literature on the linguistic features of children with hearing loss in Iran have shown a limited repertoire and lexical variety, shorter mean length utterance, delay in developing morpholo- gical and syntactic skills and a frequent use of nouns in these children (17,18).

In this study, hearing-aid users were compared with hearing children matched in ability on one aspect of language; general syntactic ability. Unlike most studies which focus on the rate of language learning or extent of delay, this study compares the syntactic profile of hearing-aid users with that of hearing children. It is known that hearing-impaired children often demon- strate delays relative to their hearing peers; this study will allow us to compare the extent of those delays across different areas of syntax acquisition. 

Given the importance of hearing sense, as discussed earlier, this study defines the importance of syntax and identifies strategies to promote the development of syntax in spoken language through listening. Further, the current research seeks to examine the syntactic skills and the role of gender in children with hearing loss under training by adopting a descriptive-analytic perspective, and compares it with the normal children in order to identify the difference between these two groups and offer effective clinical strategies for the early rehabilitative intervention by speech and language pathologists, audiologist, and linguists. 

## Materials and Methods

The present research is a cross-sectional descriptive-analytical study that draws on an available sampling method. The population included eight hearing children with an average age of 5.45 years and eight hearing-impaired children with a mean age of 5.43 years (four male children with an average age 5.45 years and four female children with an average age of 5.41 years) with bilateral sensory-neural hearing loss who had been exposed to rehabilitative programs with an auditory-verbal approach. Subjects were selected for the study after completing a questionnaire to collect personal information and recording scores, including information about personal and family status as well as auditory and training state. For children with congenital hearing loss, the information was collected from the rehabilitation file. Monolingual and Persian-speaking children with normal hearing from the same class, who were the same age as the children with hearing loss and had no precedent of hearing problems, were also included in the study. Hearing-impaired children, with normal visual and hearing parents, whose mean hearing loss was between 71 and 90 dB (severe) were included in the study. Aside from hearing impairment, exclusion criteria included history of mental disability, cerebral palsy, and a background of any other related diseases. These children were examined by a psychologist through an IQ test by Wechsler Nonverbal and Good-Enough Scales and an occupational therapist using Infant Sensorimotor Development, infant sensorimotor development and the prediction of childhood IQ developed by Hogarty S and co-workers in 1972 (30), that did not have any learning or kinesthetic problems. Accordingly, of all the potential subjects in the rehabilitation center, eight children with hearing loss were eligible for the study. This study has conducted between April–July 2013 at the rehabilitation center of cochlear implant (Shenavagostar) in Mashhad. 

The language development test, TOLDP-3, which includes syntactic subtests such as "grammatical understanding", "sentence imitation", and "grammatical completion", was used in this study. TOLDP-3 was introduced by Newcomer and Hammill in 1997 as a language development test. TOLDP-3 was developed for children aged 0–4 to 8–11 years. The test was normalized for the Iranian context by Saeid Hassan- zadeh and Asghar Minaee in 2001 (19). For the purpose of adaptation, this test was first translated into Persian and then adapted according to the Persian culture. For standardization after qualitative analysis, the test was carried out in three stages (pre-experimental, experimental, and final stages) on 1,235 preschoolers and schoolchildren (19). The present study data were collected from a manual that recorded the score of language development tests. 

Correct answers were determined by a score of 1 and incorrect answers were registered as zero. After five interval incorrect answers the test was ended.

After test completion, linguistic analysis trans- formed the data into standard scores with reference to Table A of the book based on chronological age. Then, based on the set of standard scores and with reference to Table B of the book, the syntactic gain was also calculated. Finally, after consulting the guide table for interpretation of the standard scores of the subtests, the individual syntactic performance was assessed (for detailed information, refer to the test guide handbook) (19,20).

*Statistical analysis*

Data obtained from syntactic subtests in the two classes of children with hearing loss and children with normal hearing as well as gender-segregated classes of male and female children with hearing impairment were analyzed by SPSS software based on Version 19. The mean and standard deviation were used for descriptive analysis, while the t-test was used for independent groups in the above classes because the distribution of data was normal according to the Kolmogrov-Smirnov test. P<0.05 was statistically considered significant. 

All children in the learning center have been taught by routine rehabilitation without any intervention. However, signed informed consent was provided by parents of all the children. 

## Results

According to Table 1, the mean syntactic scores of male and female children with hearing loss were 85.50 and 88.75, respectively, while this figure was 108 for children with normal hearing. With reference to Guide Table for Interpretation of Language Development Test (19) and the scores obtained from the children with normal hearing in the above table, the scores of children with hearing loss were below those for the average population (normalized standard due to percentile ranks is 90–110 score) (19).

**Table 1:** Mean gains of syntactic scores for male and female children with hearing loss and those with normal hearing

**Index** **Variable**	**Gender**	**Frequency**	**Mean** **(score)**	**Standard Deviation**
Mean gain	Girl	4	88.75	4/50
	Boy	4	85.50	6/65
	Normal	4	108	1/27

**Fig 1:**
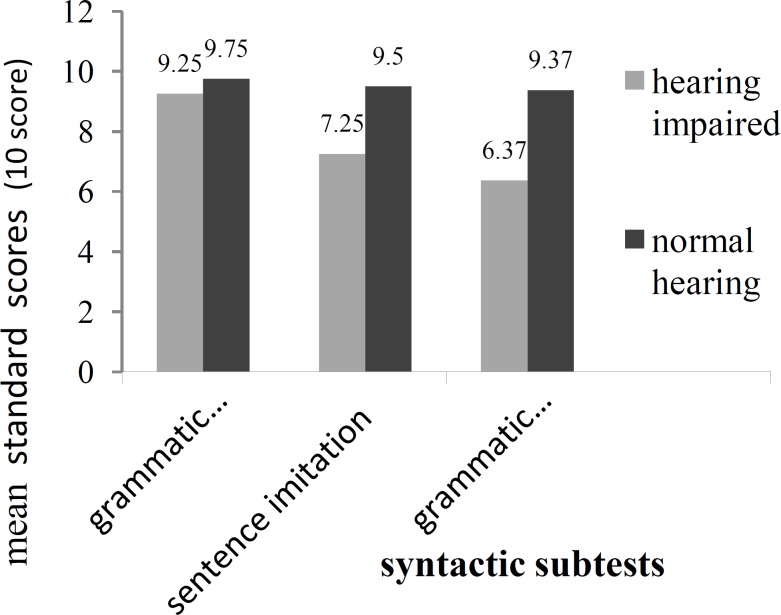
Syntactic gains of the mean standard scores in children with hearing loss and children with normal hearing

As it can be seen, there was no notable difference between the two groups in the grammatical understanding subtest, while in the two other subtests (i.e., sentence imitation and grammatical completion) a considerable difference between the standard scores of the two groups was observed.

A parametric test (t-independent) was used due to the normal distribution of the data to compare the syntactic skills of both male and female children with hearing impairment. 

**Table 2:** The Comparison of the mean of standard scores of the syntactic skills test between male and female children with hearing impairment

**Index** **Variable**	**Gender**	**N**	**Mean (score)**	**Standard deviation**	**T-test**
Grammatical Understanding	Girl	4	9/25	0/50	0/4
	Boy	4	9/25	0/95	
Sentence Imitation	Girl	4	7/5	2/38	0/322
	Boy	4	7	2	
Grammatical Completion	Girl	4	7	2	0/714
	Boy	4	5/75	2/87	
					

Table 2 indicates that the t-value of the ungrammatical understanding subtest, sentence imitation subtest, and grammatical completion was 0.4, 0.322, and 0.714, respectively, indicating that there was no significant difference between the genders in terms of grammatical understanding, Sentence imitation, or grammatical completion. 

In order to compare the syntactic skills between children with hearing loss and children with normal hearing, a parametric test (t-independent) was used due to the normal distribution of the data.

**Table 3:** Comparison of the mean of standard scores of the syntactic skills test between children with hearing loss and children with normal hearing

**Index** **Variable**	**Gender**	**N**	**Mean (score)**	**Standard deviation**	**T-test**
Grammatical Understanding	HI*	8	9/25	246	1/67
	NH**	8	9/75		
Sentence Imitation	HI	8	7/25	0/012	-2/90^*^
	NH	8	9/50		
Grammatical Completion	HI	8	6/37	0/049	-3/39^*^
	NH	8	9/37		

HI* : hearing impaired,

NH** :normal hearing

Table 3 suggests that the t-value in the grammatical understanding subtest, sentence imitation subtest, and grammatical completion subtest was 1.67,−2.90, and −3.39, respectively, indicating that there was no significant difference between hearing and hearing-impaired groups in terms of grammatical understanding but that there was a significant difference between the hearing-impaired groups in terms of sentence imitation and grammatical completion. 

## Discussion

The results of this study show that the performance of children with hearing loss and children with normal hearing in the two subtests of sentence imitation and sentence completion, which required proper use of vocabularies and sentences, were in line with the finding of Peters (21) and Bamford and Saunders (12). Peters et al found that the syntactic performance of children with hearing loss in vocabulary comprehension and usage was lower than that of children with normal hearing of the same age (22). Bamford and Saunders in their study on children with hearing loss showed that these children were more likely to use content words such as nouns and verbs in their speech, while grammatical words such as prepositions, conjunctions, and pronouns were less likely to be observed in their speech (12). Moreover, quoting Zarifian (22), Paul maintains that children with hearing loss face problems in learning and usage of lexical and functional morphemes such as adverbs, prepositions, pronouns, along with using relative clauses, complex sentences and verb inflections (23).Williams maintains that the sentences formed by people with hearing loss are simple, with frequent use of nouns and a shorter mean length utterance compared with that of people with normal hearing (23). They often have verbal errors in their speech, and their sentences are characterized by disagreement between subject and verb (24). In the present study, no significant difference was observed in grammatical understanding between children with hearing loss and children with normal hearing. On one hand, this finding is in contrast with the results of Peters concerning the vocabulary comprehension (22), and on the other hand, it is in line with the study of Yoshinaga-Itano, suggesting that timely and appropriate intervention in the early ages can promote the linguistic skills of the children with severe-to-profound hearing impairment to the level of children with normal hearing (25). Another study by Yoshinaga and Thompson revealed that the early detection of hearing impairment can help children with hearing loss with natural cognitive capacities to function almost equally to children with normal hearing of their age in all linguistic areas including phonology, morphology, syntax and pragmatics (26). 

Considering that gender is one of the factors influencing the language of the children; the studies suggest that this effect is only significant in the early language learning stages. According to the present study, there was no significant difference between male and female children with hearing impairment in syntactic aspects in all three subtests, and they had almost similar standard scores and syntactic gains. This finding is in keeping with the studies of Clark and Stewart (1973), Roberts and Block (1972), Arshi (2000) on the difference between male and female children with hearing impairment in terms of linguistic capacities, which did not report any difference in this regard (28). 

The studies of Mofidi et al show that normal students who attended preschool language learning courses had a superior performance in syntactic skills such as grammatical understanding, sentence imitation, and grammatical completion compared with students who failed to attend these classes (29). This is in keeping with this study, in which patients had been exposed to rehabilitative programs with auditory-verbal approach. The results of the present study show that the mean chronological and syntactic age of children with hearing loss were both compatible with the studies of Brown et al that assumed the language development of children with hearing loss to be identical to the language development of children with normal hearing (30).

Our study also had some limitations. Apart from limited sampling, the results were based on cross-sectional data instead of a longitudinal study. Another limitation was the range of our subjects with hearing loss, since all subjects had severe hearing loss. To investigate the hearing loss effect it also could be appropriate to evaluate cochlear implant.

## Conclusion

 Syntax is one of the skills that is learned during growth, particularly the critical period. The development of language skills is influenced by the manner of speech of the people around the child, especially the mother, as well as sentence complexity, repetition and practice, and proper communicative events. Thus, the comparison of the syntactic skills of children with hearing loss and children with normal hearing in this paper shows that the earlier exposure of children with hearing loss to natural and appropriate environment as well as timely and proper rehabilitative education with emphasis on auditory sense can improve the performance of these children to the level of normal children in some syntactic areas, and minimize the delay in other areas through persistency and proper planning. 

We recommend that, in the future, studies include a large sample and a specific intervention. After a period of time, studies should also investigate children with a hearing aid with respect to other aspects of language skills such as speech production and speech perception.
